# Checkpoint blockade and the stem‐like T cell trade‐off

**DOI:** 10.1002/1878-0261.70257

**Published:** 2026-04-20

**Authors:** Julie M. Mazet, Johanna A. Joyce

**Affiliations:** ^1^ Department of Fundamental Oncology University of Lausanne Switzerland; ^2^ Ludwig Institute for Cancer Research University of Lausanne Switzerland; ^3^ Agora Cancer Research Centre Lausanne Switzerland

**Keywords:** cancer immunity, immune checkpoint blockade, Stem‐like T cells

## Abstract

Stem‐like T cells are central to the efficacy of programmed cell death protein 1 (PD1) blockade, sustaining long‐term immune responses by serving as a renewable reservoir for anti‐tumor effector CD8+ T cells. However, the mechanisms governing their maintenance and regulation in cancer remain incompletely understood. Addressing this gap, Hor *et al*. combined high‐dimensional 3D‐imaging with immunological profiling to define the niche of stem‐like T cells within tumor‐draining lymph nodes in murine cancer models. Their study identifies a critical role for conventional type 1 dendritic cells (cDC1s) and the PD1 pathway in preserving high‐affinity tumor‐specific stem‐like T cells. cDC1s deliver sustained T‐cell receptor (TCR) stimulation together with PD‐L1/2 co‐inhibitory signals that support stemness, proliferation, and survival. Strikingly, disruption of PD1 signaling transiently enhances effector T cell expansion but promotes differentiation and apoptosis of stem‐like T cells, ultimately depleting this essential pool. These findings reveal a potential long‐term vulnerability of immune checkpoint blockade, particularly when tumors are not eradicated during the initial treatment response.

## Graphical abstract

Stem‐like T cells are key to the success of programmed cell death protein 1 (PD1) blockade, as they sustain long‐term anti‐tumor response by continuously generating effector CD8+ T cells. However, how these cells are maintained in cancer is not fully understood. Hor *et al.* used advanced 3D imaging and immunological analyses to study their niche in tumor‐draining lymph nodes. They show that conventional type 1 dendritic cells (cDC1s) and PD1 signaling work together to preserve high‐affinity stem‐like T cells by providing ongoing stimulation alongside inhibitory signals. While PD1 blockade boosts effector CD8+ T cell expansion in the short term, it also drives stem‐like T cells to differentiate or die, depleting this critical reservoir for long‐term response. These findings highlight a potential limitation of immune checkpoint therapies, especially when tumors are not fully cleared after the initial treatment.

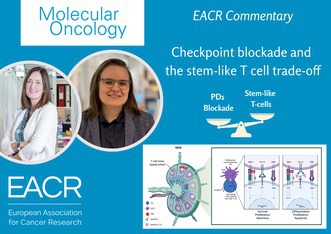

**Abbreviations**
cDC1: Type 1 conventional dendritic cells
ICB: Immune checkpoint blockade
PD1: Programmed cell death protein 1
PD‐L1/2: Programmed death‐ligand 1 and 2
TCR: T‐cell receptor
tdLN: tumor‐draining lymph nodes

Stem‐like T cells, also termed progenitor exhausted T cells, represent a pool of resource cells that sustains ongoing immune responses in chronic settings such as persistent infection, autoimmunity, and cancer [1, 2]. These CD8+ T cells retain self‐renewal capacity, enabling long‐term persistence while continuously generating differentiated progeny, including cytotoxic effector CD8+ T cells that kill target cells such as infected cells or tumor cells [3]. Stem‐like T cells were first described in 2016 by Im *et al*. [4] as the subset responsible for the proliferative response to blockade of programmed cell death protein 1 (PD1) in a chronic infection model driven by lymphocytic choriomeningitis virus. Focusing on virus‐specific CD8+ T cells, the authors and others identified a quiescent CD8+ T cell population that arises specifically during chronic antigen exposure, in contrast to acute infection models [5]. Upon stimulation, stem‐like T cells differentiate into early‐exhausted effector CD8+ T cells with strong proliferative capacity, a feature that is essential for the efficacy of anti‐PD1 treatment [6]. This foundational observation also implied that PD1 signaling is integral to their biology [7]. Since this initial description a decade ago, growing attention has focused on stem‐like T cells, particularly in cancer, to define their dynamics with the goal of improving immunotherapy strategies. Stem‐like T cells have been shown to reside in lymphoid tissues [8, 9] and can traffic to tumor sites [10]. Overall, it is speculated that stem‐like T cells persist in specific niches at low frequencies within the CD8+ T cell population in chronic contexts. However, critical questions remain about how, when, and where they arise. How is their self‐renewal capacity maintained *versus* their differentiation being driven? Do they have other functions beyond serving as a resource pool? And how can we manipulate their presence and persistence in tumors?

Addressing several of these questions, Hor *et al*. [11] investigated stem‐like T cell fitness in cancer, focusing on how a subset of tumor‐specific CD8+ T cells can maintain a stem‐like state despite chronic antigen stimulation, a major driver of exhaustion. They further examined whether the emergence of stem‐like T cells in lymph nodes is restricted to an early priming event or instead continues dynamically over time.

## 1 Stem‐like T cells thrive in cDC1 niches

**Fig. 1:**
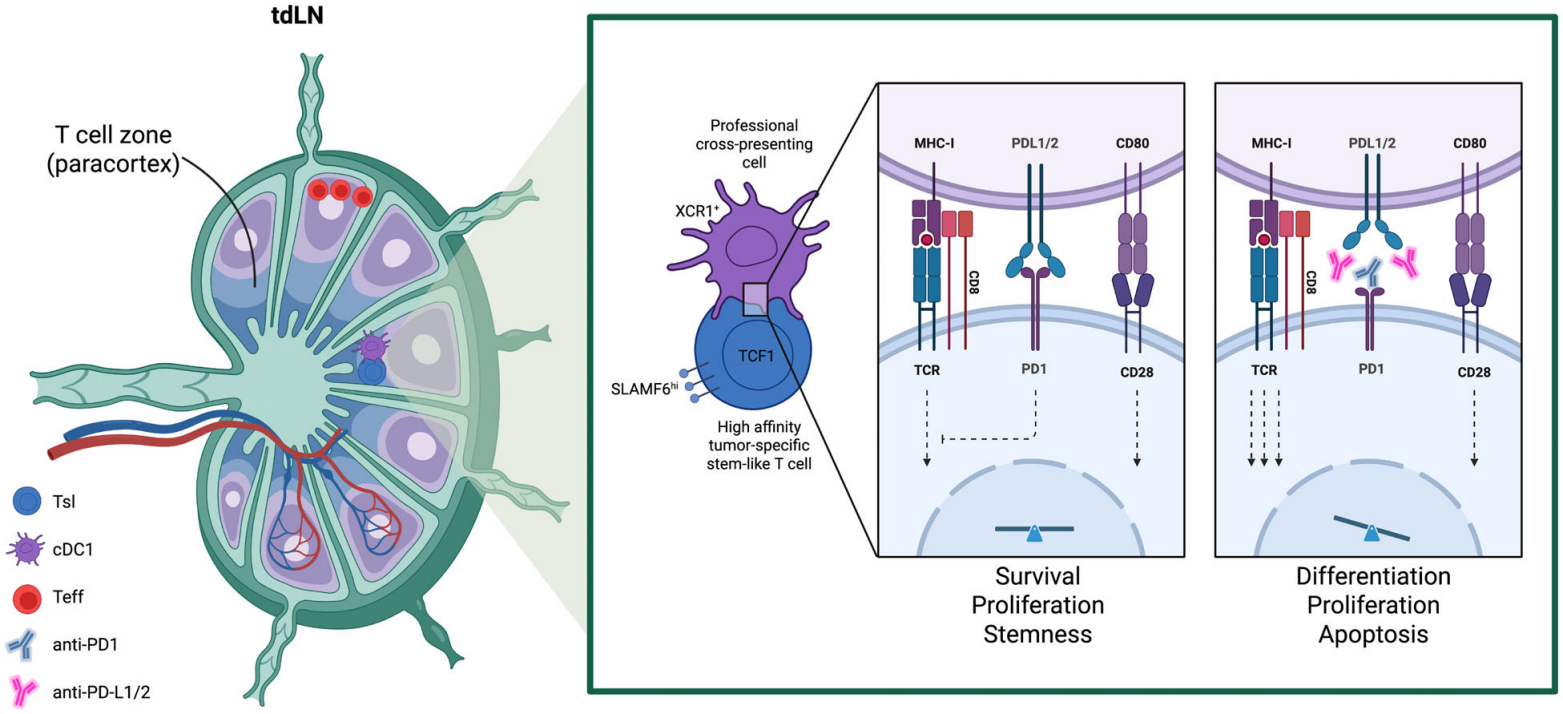
Schematic representation of the key findings from Hor et al. Nature 2026. This image shows the interaction between high‐affinity stem‐like T cells (TCF1 + SLAMF6+) and cDC1 (XCR1+) in the T cell zone of the tumor‐draining lymph node (tdLN) in a murine cancer model. It depicts the essential role of the PD1 inhibitory axis in counteracting sustained TCR signaling during late antigen presentation, thereby regulating the fate of stem‐like T cells. Teff = effector T cells, Tsl = stem‐like T cells, cDC1 = type I conventional dendritic cells. Figure created with Biorender.

To define where tumor‐specific stem‐like T cells localize, the authors took advantage of the OVA – OT‐I/tetramer model, incorporating the model antigen ovalbumin (OVA) and OVA‐TCR I clone CD8+ T cells (OT‐I). Briefly, they used mice harboring OVA‐expressing Kras^G12D/+^Trp53^−/−^ lung adenocarcinoma cells, intradermally implanted into immunocompetent recipients, and either adoptively transferred naïve OVA‐specific CD8+ T cells (OT‐I) or used the OVA‐tetramer technology to track endogenous tumor‐specific CD8+ T cells. Tumor‐draining lymph nodes (tdLN) were collected and analyzed using a high‐dimensional 3D tissue imaging approach in parallel with conventional flow cytometry to map the CD8+ T cell landscape. This strategy revealed that stem‐like OT‐I cells localize within the T cell zone of the tdLN (Fig. 1), where they form clusters with cDC1s, the Type 1 conventional dendritic cells specialized for cross‐presentation to CD8+ T cells. Notably, analogous stem‐like T cells/cDC1 clusters were also observed during endogenous polyclonal CD8+ T cell responses.

To probe whether these spatially organized clusters reflect ongoing antigen recognition, the authors quantified NFAT nuclear translocation as a readout of active T‐cell receptor (TCR) signaling. Strikingly, stem‐like T cells that clustered with cDC1 in tdLN maintained TCR‐triggered activity days beyond the initial priming phase, even after substantial proliferation. This indicates that stem‐like T cells can self‐renew rather than obligatorily differentiate, despite sustained TCR stimulation, underscoring a potential survival and fate‐shaping role for continued antigen recognition. In this context, while some cells progress along an exhaustion trajectory, others appear relatively protected from terminal differentiation; in both cases, the cells can persist for prolonged periods.

The authors further observed that stem‐like T cells undergoing late antigen presentation are preferentially retained within tdLN (Fig. 1), while stem‐like T cells with lower PD1 expression and correspondingly lacking evidence of late antigen presentation are also detected at tumor sites. Consistent with a functional requirement for cDC1s beyond priming, ablation of cDC1s after the priming phase resulted in a marked loss of tumor‐specific stem‐like T cells, revealing a critical role for cDC1‐derived survival signals within tdLN niches.

## 2 TCR‐PD1 signaling: A fine‐tuned equilibrium

Having established that stem‐like T cells preferentially cluster with cDC1 in tdLN well after initial priming, Hor *et al*. next asked whether stem‐like fate is dictated primarily by intrinsic properties of CD8+ T cells, or instead by extrinsic positioning within cDC1 niches. A key parameter to consider was TCR affinity, given extensive evidence across diverse contexts that TCR signal strength shapes CD8+ T cell fate decisions [12]. To interrogate this, the authors considered three parameters: (a) OVA‐tetramer binding normalized to expression of the co‐receptor CD3, representing an imputed TCR affinity index; (b) PD1 expression as a proxy for activation strength; and (c) SLAMF6 expression to define the stem‐like T cell phenotype. Within tdLN, higher‐affinity CD8+ T cells are selectively enriched in the stem‐like compartment. Moreover, among stem‐like T cells, those with the highest imputed TCR affinity exhibited the highest PD1 expression, consistent with strong TCR engagement.

These findings support a model in which late antigen presentation within tdLN niches promotes the retention and selection of high‐affinity stem‐like T cell clones (Fig. 1), thereby sustaining affinity maturation at the population level and enabling ongoing generation of high‐affinity effector CD8+ T cells. However, they also underscore a central paradox: in many contexts, sustained TCR triggering coupled to proliferation would be expected to drive effector differentiation rather than preserve a memory‐like, stem‐like state [13].

Given that PD1 is upregulated upon activation and functions as an inhibitory checkpoint, the authors next examined the spatial organization of PD1 on CD8+ T cells and the programmed death‐ligands 1 and 2 (PD‐L1 and PD‐L2) within tdLN. Using 3D imaging, they showed that PD1 and PD‐L1 localize to immunological synapses between CD8+ T cells and cDC1s; flow cytometry further confirmed that cDC1s express both PD1 ligands. To test whether PD1 pathway engagement preserves stem‐like T cell fitness, the authors co‐blocked PD‐L1 and PD‐L2 *in vivo*. This intervention increased the proliferation of tumor‐specific CD8+ T cells, reduced stemness potential among cells clustered with cDC1, and increased apoptosis overall. Together, these results indicate that potent antigenic stimulation in the absence of PD1‐mediated restraint destabilizes the CD8+ T cell ecosystem, promoting activation‐induced cell death in the stem‐like compartment.

Importantly, these findings raise concerns about potential long‐term consequences of what is typically considered an “effective” immune checkpoint blockade (ICB) response: high‐affinity stem‐like T cells may lose stemness, differentiate toward effector/exhausted states, and undergo apoptosis—thereby depleting a critical reservoir of resource cells required for durable immunity.

## 3 The biological downside of ICB

Consistent with this model, PD1 axis blockade led to fewer stem‐like T cells and a shift toward lower TCR affinity within the tdLN stem‐like pool after 8 days of treatment, suggesting that PD1 pathway disruption reshapes clonal composition. Mechanistically, if high‐affinity stem‐like T cells are driven either toward effector differentiation (representing the short‐term therapeutic benefit of ICB) or toward apoptosis via activation‐induced cell death, this may open “niche space” for lower‐affinity clones to access cDC1 niches and undergo selective expansion. At the population level, PD1 blockade thus reveals a measurable shift in TCR affinity, highlighting divergent responses across CD8+ T cell subsets.

Notably, discontinuation of PD1 blockade did not restore the high‐affinity stem‐like T cell pool in tdLN over time, suggesting that loss of stemness potential may be effectively irreversible and not readily compensated by replacement from elsewhere. Moreover, PD1 signaling constrained CD8+ T cell expansion within antigen‐presentation niches independently of CD4+ T cells, as shown by depletion experiments. Overall, blocking PD1‐PDL1/2 interactions dramatically altered the TCR clonality landscape, producing a burst of effector proliferation that could not be reproduced once high‐affinity stem‐like T cells were lost.

## 4 Conclusions

This study highlights a fundamental trade‐off whereby potent PD1 blockade can enable tumor control but depletes high‐affinity stem‐like T cells, whereas weaker inhibition might preserve these resource cells but at the expense of immediate treatment efficacy. More broadly, TCR triggering can produce distinct outcomes, including survival, activation, proliferation, differentiation, or cell death, depending on signal strength and the surrounding environment. Therefore, maintaining a tightly regulated balance is essential. PD1 upregulation is a physiological component of CD8+ T cell activation, acting as a natural brake that dampens excessive TCR signaling. In addition, soluble cues are critical in shaping productive CD8+ T cell responses and permitting different clonal populations to expand. Stem‐like T cells appear particularly sensitive to their microenvironment; they reside within specialized niches and respond to soluble cues such as IFNg [14], and IL‐10 [15], which can profoundly influence their maintenance and fate. Collectively, this highlights cellular plasticity and the integration of a broad spectrum of environmental activating and inhibitory inputs, the balance of which ultimately determines cell fate.

Several questions now follow. Because stem‐like T cells arise in chronic settings in which antigen persists, they resemble an immune memory‐like state, yet are not considered to represent canonical immune memory. It will therefore be informative to examine these cells in a setting where the tumor has been eliminated. Do the cells persist systemically, and how does the loss of tumor antigen reshape their abundance and phenotype? Can lower‐affinity stem‐like T cells evolve toward higher affinity over time, particularly in the context of ongoing tumor evolution? Finally, assessing stem‐like T cell fitness in orthotopic and metastatic models will be critical to understand how anatomical context, antigen presentation, and niche architecture collectively shape durable anti‐tumor immunity.

## Conflict of interest

The authors declare no conflict of interest.

## Author contribution

J.M.M. and J.A.J. wrote the manuscript. J.M.M. prepared the figure.
